# Evaluation of metallic trace elements contents in some major raw foodstuffs in Burkina Faso and health risk assessment

**DOI:** 10.1038/s41598-022-08470-z

**Published:** 2022-03-15

**Authors:** Bazoin Sylvain Raoul Bazié, Muller Kiswendsida Abdou Compaoré, Moumouni Bandé, Stephane Dissinviel Kpoda, Naamwin-So-Bawfu Romaric Méda, Tebkieta Marceline Ouedraogo Kangambega, Inoussa Ilboudo, Barkissa Yonaba Sandwidi, Fulbert Nikiema, Alphonse Yakoro, Imaël Henri Nestor Bassolé, Hervé Hien, Elie Kabré

**Affiliations:** 1 Laboratoire National de Santé Publique (LNSP), 09 BP 24 Ouagadougou 09, Burkina Faso; 2 Laboratoire de Biologie Moléculaire, d’Epidémiologie Et de Surveillance Des Agents Transmissibles Par Les Aliments (LaBESTA), Centre de Recherche en Sciences Biologiques Alimentaires Et Nutritionnelles (CRSBAN), École Doctorale Sciences Et Technologies, Université Joseph KI-ZERBO, 03 BP 7021 Ouagadoudou 09, Burkina Faso; 3 Laboratoire de Biochimie de L’UFR/SDS, Université Joseph KI-ZERBO, 03 BP 7021 Ouagadoudou 09, Burkina Faso; 4 Université Joseph KI- ZERBO, Centre Université de Ziniaré, 03 BP 7021 Ouagadougou 03, Burkina Faso; 5 Institut National de Santé Publique, BP 10278, Ouagadougou, Burkina Faso

**Keywords:** Biochemistry, Metals

## Abstract

Diet based on cereal, vegetables, oleaginous and dried fish are providing essential metallic elements. It can be also a source of exposure to toxic metallic elements. The aims of this study were to evaluate the contents on nine metallic trace elements (Fe, Zn, Mn, Co, Cd, Pb, Cu, Ni, Cr) in some major raw foodstuffs including rice, maize, peanut, tomato and dried fish in Burkina Faso and assess the health risk of these elements. Two hundred twenty-two samples were collected and analyzed by atomic absorption spectrometry. The health risk assessment was based on the United States Environment Protection Agency (USEPA) model. Iron and Zinc were the elements with the highest concentrations in the investigated foodstuffs. The iron highest median value (68.80 mg/kg) was observed in dried fish followed by maize (43.09 mg/kg) and peanuts (28.92 mg/kg). Rates of 77.95%, 66.66% and 32.5% obtained respectively fro tomato, maize and rice samples were above the maximum limit of lead set by Codex Alimentarius while 47.6%, 71.16% and 0% of maize, tomato and rice samples respectively have shown concentration above the maximum limit of cadmium. Chromium had shown higher contribution rate to the maximum daily intake of 167.11%, 34%, 2% and 8.53% for rice, maize and peanut respectively. A non-cancer risk situation has been observed on rice, maize and peanut consumption. None of the index risk values was above the threshold set by USEPA.

## Introduction

Food may be the primary route of exposure to contaminants from multiple chemical classes. Therefore, food safety is a major public health concern and its demand by consumers worldwide has stimulated research regarding the risk associated with consumption of foodstuffs contaminated by various contaminants^1^.

Among the chemical contaminants recognized as potential food threats, metallic trace elements (MTE) are of great concern for human health.

The term Trace elements is used for elements existing in natural and perturbed environments in small amounts, whose excessive bioavailability has a toxic effect on the living organism^2^.

There are two classes of trace elements, essential and non-essential metals. Elements such as copper (Cu), manganese (Mn), selenium (Se) and zinc (Zn) also called micronutrients are essential for living organism and play important roles in the functioning of the critical enzyme system^3^. They are present in trace quantities; however, high intake of these elements could also cause health damage^4^. Whereas, heavy metals like cadmium (Cd), chromium (Cr), mercury (Hg), lead (Pb) arsenic (As) generally refer to metals having densities greater than 5 g.cm^-3^.^5^ have no established biological functions, and are considered as non-essential metals^6^.

Trace elements can occur as residues in foodstuffs through their presence in the environment as results of human activities such as industry, farming and car exhaust^7^. Foodstuffs can also be contaminated during their process, storage and marketing. The food sold in the open market can easily accumulate a high level of heavy metals^8^.

Due to their non-biodegradability and toxicity at low concentration, MTE intake through the food chain is a problem receiving increasing attention^9^.

The USEPA and the International Agency for Research on Cancer (IARC) have classified some elements as either “known” or “probable” human carcinogens based on epidemiological and experimental studies showing an association between exposure and cancer incidence in humans and animals^10^.

The diet in Burkina Faso is based on staple foods, mainly cereals, legumes, roots, and tubers^11^. According to the high frequency consumption of these food groups^12^, cereal (maize and rice), legume (tomato), oleaginous (peanut) and dried fish have been selected as major consumed food items. Although these foodstuffs are an important source of a wide range of essential trace elements for humans. They can also carry toxic metals. Number of studies have been reported on the MTE contents in cereal, oleaginous, legumes and fish^13–17^ in over the world. However, there are few data on MTE contamination in these food items in Burkina Faso. Furthermore, the risk assessment of dietary exposure remains unknown. Therefore, the objectives of this study were to (1) evaluate the contents of Iron, Zinc, Cadmium, Cobalt, Cupper, Lead, Manganese, Nickel, Silver and Chromium in maize, rice, peanut and tomato, (2) estimate daily MTE intake through consumption of these food and (3) determine the cancer and non-cancer risks associated with the MTE intake using determinist risk assessment approach.

## Material and methods

### Sampling

The sampling was carried out across the following locations: Ouagadougou, Bobo-Dioulasso, Niangoloko, Dakola and Cinkanse. The samples were collected from different open markets located in these cities. A total of 222 samples including 40 samples of rice, 19 samples of maize, 59 samples of peanut, 59 samples of tomato and 45 samples of dried fish were collected from 2 November to 3 October 2020. The dried samples were kept at room temperature while tomato sample were frozen prior for analyses.

### Reagents and standards

The de-ionized water was obtained from the water purification system Lab Tower Aft (Thermo scientific, Niederelbert, Germany). Nitric and chlorhydric acids were provided by Hiperpur, Panreac AppliChem, Darmstadt, Germany and the individual stock standard solutions containing 1000 mg.L^-1^ purchased from MERK (Darmstadt, Germany).

### Apparatus

RassMill from Romerlab (Austria) was used for grinding cereal samples and the stainless-steel blender (Mixer Grinder HL7810/00, India) for peanut and tomato samples. The sample digestion was carried out with the Techne heat block (DB-2P-Techne, Staffordshire, United Kingdom). The elementary analyses were performed by an atomic absorption spectrometer VARIAN 240FS (Mulgrave, Australia).

### Samples preparation and analysis

HNO3/HCl (3:1) mixture was used for the sample digestion as described by Demirel et al.^18^. Briefly 0.5 g of sample was digested in a test tube by 5 ml of the acid mixture at 150 °C for 2½ hours. The volume of the digestate was completed to 100 ml with de-ionized water and filtered. The analysis was done with atomic absorption spectrometry on flame mode with external calibration curve.

The quality control of analytical performance was apply as described by Bazié et al.^19^.

### Exposure assessment

The USEPA human health risk assessment model was applied^1,9^. The average daily dose (ADD) (mg/kg/day) of metals was determined as follow:^20–22^

$$ ADD = \frac{C \times IQ \times EF \times ED}{{BW \times AT }} \times CF $$

where ADD is the average daily dose ingested (mg/kg/day), C is the concentration of MTE in food items (mg/kg), IR is the ingested quantity of food (kg/day); EF is the exposure frequency (days/year), ED is the exposure duration (years), BW is the average body weight (kg), AT is the averaging time (days), CF is a conversion factor.

### Non-carcinogenic risk assessment

The ratio of the average daily dose and the reference dose (RfD) of the MTE expressed as the hazard quotient (HQ) was used^20,21^.

$$ HQ = { }\frac{ADD}{{RfD}} $$

where HQ is the hazard quotient of the individual trace element (mg/kg/day); and RfD is the risk oral reference dose of the concern element (mg/kg/day).The following RfD : 1.5, 0.04, 0.004, 0.7, 0.3, 0.0003, 0.014, 0.001 and 0.011 have been used for Cr, Cu, Pb, Fe, Zn, Co, Mn, Cd, Ni respectively^23,24^.

To assess multiple MTE exposure, the non-carcinogenic risk is expressed as the hazard index (HI), which is the sum of the HQ^21,25^:

$$ HI = \mathop \sum \limits_{i = 1}^{n} HQ $$

### Carcinogenic risk assessment

The carcinogenic risk was determined according to the following equation:^20,21^

$$ RISK = ADD \times SCF $$

where CSF is the carcinogenic slope factor (mg/day/kg), attributed to the MTE having a carcinogenic property.

### Statistical analysis

The SPSS package version 23.0.0.0. was used for statistical analysis. The data were check for normal distribution. The non-parametric tests have been performed.

### Institutional review board

The design and conduct of the survey were approved by the Institutional Ethics Committee Board of the Ministry of Health of Burkina Faso since the experimental research on plants complied with the relevant institutional, national, and international guidelines and legislation.

## Results and discussion

### Metallic trace elements contents

Since the data failed to show a normal distribution, the metallic trace elements contents are presented in Tables 1 and 2 by the range of concentration and the median value with the interquartile range.

**Table 1:** Metallic trace elements contents in cereals (mg/kg).

Samples	Sites		Pb	Zn	Cd	Ni	Co	Mn	Fe	Cr	Cu
Rice	Ouagadougou	Range	< LD-3.90	10.72–18.44	< LD-0.14	1.10–5.69	0.64–3.34	1.92–9.06	7.86–32.70	1.54–2.69	0.27–5.28
		Med	0.10 (0.15)	14.38 (4.10)	0.08 (0.06)	1.50 (0.95)	0.88 (0.35)	4.08 (2.91)	11.78 (5.00)	2.02 (0.30)	3.03 (1.00)
	Bobo Dioulasso	Range	< LD-2.77	10.72–17.49	0.04–0.10	1.10–1.52	0.64–0.89	2.16–9.06	10.26–32.70	1.54–2.12	0.27–4.12
		Med	0.14 (0.1)	14.02 (5.14)	0.07 (0.05)	1.42 (0.2)	0.83 (0.12)	3.64	14.46 (11.03)	1.84 (0.4)	2.95 (1.11)
	Niankologo	Range	< LD-2.01	10.72–15.70	< LD-0.14	1.42–2.30	0.83–1.35	1.92–9.06	8.48- 32.70	1.86–2.06	2.9–05.28
		Med	0.18 (0.24)	13.22 (3.38)	0.12 (0.04)	1.56 (0.5)	0.91 (0.29)	4.48 (5.40)	11.52 (7.4)	2.06 (0.16)	3.20 (0.44)
	Dakola	Range	< LD-3.56	12.48–18.44	< LD-0.14	1.42–2.30	0.83–1.35	1.92–7.56	10.26–23.42	1.84–2.12	0.27–4.12
		Med	0.26 (0.93)	14.50 (3.41)	0.08 (0.06)	1.62 (0.52)	0.83 (0.3)	3.59 (5.20)	12.74 (5.54)	2.02 (0.16)	0.27 (3.28)
	Cinkasse	Range	< LD-0.18	11.70–17.49	0.04–0.10	1.42–1.92	0.83–1.12	4.98–12.68	8.48–15.88	1.54–2.69	2.14–3.28
		Med	0.16 (0.09)	15.70 (5.03)	0.06 (0.04)	1.42 (0.32)	0.83 (0.18)	9.06 (4.81)	11.52 (4.96)	2.06 (1.07)	2.90 (0.83)
	Total	Range	< LD-3.90	10.72–16.44	< LD-0.14	1.10–5.69	0.64–3.34	1.92–12.68	7.86–32.70	1.54–2.69	0.27–5.28
		Med	0.15 (015)a	13.92 (3.65)a	0.08 (0.05)	1.50 (0.26)a	0.88 (0.15)a	4.48 (4.86)a	12.04 (5.10)a	1.98 (0.26)a	3.00 (1.11)a
Maize	Ouagadougou	Range	< LD-0.78	12.36–23.43	< LD-0.24	0.14–1.04	1.01–1.72	< LD-4.61	49.76–96.27	0.02–0.15	1.66–3.93
		Med	0.23 (0.53)	18.06 (8.28)	< LD	0.67 (0.67)	0.39 (0.4)	3.23 (3.3)	79.85 (37.64)	0.11 (0.1)	2.36 (1.34)
	Bobo Dioulasso	Range	< LD-0.46	6.84–14.85	< LD-0.48	< LD-1.86	0.22–1.15	< LD13.77	38.54–53.69	< LD-0.30	1.66–3.12
		Med	0.38 (0.38)	8.25 (6.51)	0.12 (0.42)	0.45 (1.46)	0.26 (0.86)	1.60 (10.75)	43.09 (12.53)	0.01 (0.23)	2.07 (1.13)
	Niankologo	Range	< LD-1.48	7.69–22.39	< LD-0.50	0.47–1.73	0.27–1.91	< LD11.46	62.01–189.60	< LD-0.22	1.57–3.48
		Med	0.46 (0.97)	15.40 (10.98)	0.25 (0.49)	0.99 (1.12)	0.57 (0.66)	7.14 (9.58)	69.90 (78.40)	0.10 (0.21)	2.19 (1.1)
	Dakola	Range	< LD-0.31	9.26–20.98	< LD-0.25	0.59–1.42	0.22–1.67	2.46 (5.88)	53.69–109.07	< LD-0.14	2.00–2.21
		Med	0.15 (0.42)	15.12 (-)	0.12 (-)	1.01 (-)	0.94 (-)	4.17 (-)	81.38 (-)	0.07 (-)	2.10 (-)
	Cinkasse	Range	< LD-0.46	6.84–7.25 (-)	< LD-0.48	0.32–1.86	0.22–0.58	0.731 (3.77)	40.78–45.41	< LD-0.30	2.14–3.12
		Med	0.23 (-)	7.05	0.24 (-)	1.09 (-)	0.40 (-)	7.25 (-)	43.09 (-)	0.15 (-)	2.63 (-)
	Total	Range	< LD-1.48	6.84–23.43	< LD-0.50	< LD-1.86	< LD-1.09	< LD-13.77	38.54–189.60	< LD-0.30	1.57–3.93
		Med	0.31 (0.46)b	13.60 (0.30)a	< LD)	0.69 (1.05)b	0.40 (0.61)b	2.85 (1.10)b	58.49 (40.97)b	0.08 (0.16)b	2.16 (0.64)a

**Table 2:** Metallic trace elements contents in tomato, peanuts and dried fish (mg/kg).

Samples			Pb	Zn	Cd	Ni	Co	Mn	Fe	Cr	Cu
Tomato	Ouagadougou	Range	< LD-2.10	1.38–10.82	< LD-0.27	0.50–2.85	0.3–1.67	0.76–8.04	1.34–17.83	0.75–1.36	0.09–1.24
		Med	1.55 (1.66)	5.57 (4.18)	0.05 (0.02)	0.69 (2.19)	0.4 (1.29)	1.17 (5.75)	5.86 (2.2)	1.03 (0.23)	0.77 (0.83)
	Bobo Dioulasso	Range	< LD-1.30	2.33–8.79	0.04–9.00	0.49–1.23	0.28–0.73	0.87–1.95	5.66–10.81	< LD-0.63	0.26–0.83
		Med	0.80 (0.38)	4.64 (3.37)	0.09 (0.04)	0.96 (0.37)	0.56 (0.21)	1.46 (0.62)	8.30 (3.44)	0.21 (0.48)	0.49 (0.29)
	Niankologo	Range	< LD-0.90	1.76–4.46	< LD-0.14	1.10–1.56	0.64–0.91	1.09–2.54	4.96–12.85	0.56–1.30	0.22–0.89
		Med	0.45 (0.6)	2.39 (1.56)	0.12 (0.05)	1.18 (0.26)	0.69 (0.15)	1.71 (0.77)	7.31 (5.12)	0.95 (0.42)	0.44 (0.3)
	Dakola	Range	< LD-1.10	2.38–5.93	< LD-0.16	1.23–1.70	0.72–1.00	1.29–2.15	4.96–10.95	1.28–1.65	0.40–0.79
		Med	0.40 (0.85)	4.05 (1.67)	0.15 (0.06)	1.46 (0.29)	0.85 (0.17)	2.04 (0.54)	7.52 (3.19)	1.46 (0.21)	0.56 (0.13)
	Cinkasse	Range	< LD-1.30	2.83–10.82	< LD-9.00	0.49–2.85	0.28–1.67	0.87–8.04	1.34–10.28	< LD-1.69	0.10–0.83
		Med	0.80 (0.8)	5.88 (3.54)	0.09 (0.04)	0.92 (0.9)	0.54 (0.52)	1.37 (1.07)	7.13 (4.19)	0.19 (0.93)	0.46 (0.36)
	Total	Range	< LD-2.10	1.38–10.82	< LD-9.00	0.49–2.85	0.28–1.67	0.76–8.04	1.34–17.83	< LD-1.69	0.09–1.24
		Med	0.80 (1.10)a	4.39 (3.23)a	0.09 (0.07)a	1.09 (0.68)a	0.64 (0.4)a	1.50 (0.9)a	7.10 (3.70)a	0.95 (0.86)a	0.52 (0.40)a
Dried fish	Ouagadougou	Range	< LD-5.80	1.44–17.31	< LD-0.54	0.40–5.65	0.23–3.32	8.30–14.52	40.30–204.82	0.23–3.09	0.15–0.67
		Med	0.80 (2.21)	7.21 (5.77)	0.08 (0.1)	4.17 (3.43)	2.45 (2.01)	12.52 (2.06)	60.38 (26.08)	1.87 (0.76)	0.18 (0.04)
	Bobo Dioulasso	Range	0.22–3.20	6.28–17.31	< LD-0.12	0.57–5.61	0.33–3.30	8.30–14.52	49.14–178.98	0.89–2.67	0.15–0.67
		Med	0.97(2.04)	10.10 (6.36)	0.08 (0.06)	3.28 (3.51)	1.92 (2.06)	13.32 (1.67)	64.18 (33.56)	1.87 (0.52)	0.18 (0.11)
	Niankologo	Range	0.2–2.60	2.88–17.31	< LD-0.54	0.40–5.61	0.23–3.30	8.30–14.38	56.34–113.60	0.89–2.67	0.15–0.67
		Med	1.40 (1.98)	6.74 (6.00)	0.09 (0.05)	4.05 (3.15)	2.38 (1.85)	13.06 (2.97)	61.43 (7.54)	1.87 (0.5)	0.18 (0.04)
	Dakola	Range	< LD-1.77	1.44–15.87	< LD-2.01	0.40–5.65	0.23–3.32	11.22–14.38	49.14–199.78	0.23–3.09	0.18–0.34
		Med	1.40 (0.8)	4.32 (8.66)	0.1(1.09)	3.89 (5.02)	2.28 (2.95)	13.46 (2.06)	64.17 (37.22)	1.83 (1.89)	0.18 (0.03)
	Cinkasse	Range	< LD-2.40	4.32–15.87	0.08–2.07	3.28–5.65	1.19–3.32	11.22–14.06	57.56–106.60	0.89–2.09	0.18–0.23
		Med	1.30 (1.69)	8.65	0.1 (1.49)	5.60 (1.79)	3.29 (1.05)	12.69	68.80 (40.99)	1.69 (0.99)	0.20 (0.04)
	Total	Range	< LD-5.80	1.44–17.31	< LD-2.07	0.40–5.65	0.23–3.32	8.30–14.52	40.30–204.82	0.23–3.09	0.15–0.67
		Med	1.20 (2.00)b	7.21 (5.77)b	0.08a (0.04)	4.17 (3.43)b	2.45 (2.01)b	13.32 (2.06)b	62.48 (27.22)b	1.87 (0.58)b	0.18 (0.36)a
Peanuts	Ouagadougou	Range	< LD-5.80	1.45–39.15	< LD-0.54	2.96–5.49	1.74–3.22	11.38–34.04	4.44–380	1.70–2.82	0.26–13.36
		Med	0.80 (1.2)	5.83 (14.94)	0.12(0.10)	5.31 (1.14)	3.12 (0.67)	19.52 (7.57)	25.14 (16.71)	2.67 (0.27)	0.29 (8.75)
	Bobo Dioulasso	Range	< LD -2.20	32.28–61.37	< LD-0.44	3.06–8.54	1.80–5.02	17.94–25.64	26.62–188.44	1.45–3.86	0.78–9.54
		Med	0.60 (1.00)	39.00 (9.92)	0.27 (0.08)	5.53 (2.42)	3.25 (1.42)	20.53 (7.70)	31.25 (19.98)	2.47 (1.83)	10.47 (0.99)
	Niankologo	Range	< LD -1.8	13.81–37.44	0.22–0.48	4.74–7.12	2.78–4.18	8.60–19.44	15.88–104.82	1.23–3.19	4.58–10.56
		Med	0.6 (1.2)	31.02 (6.35)	0.28 (0.12)	5.32 (1.26)	3.12 (0.74)	17.70 (2.54)	26.80 (18.16)	2.02 (0.62)	9.74 (1.96)
	Dakola	Range	< LD-1.00	4.90–35.87	< LD-0.34	1.77–5.64	1.04–3.31	4.66–25.43	7.70–61.40	1.87–5.40	0.67–9.94
		Med	0.6 (0.6)	30.96 (8.46)	0.26 (0.13)	3.58 (3.40)	2.10 (2.00)	18.28 (12.75)	56.00 (36.94)	4.98 (3.39)	7.90
	Cinkasse	Range	< LD-0.60	21.30–41.71	< LD-0.42	4.04–6.68	2.37–3.92	15.22–22.14	22.30–151.30	1.18–4.62	5.86–11.76
		Med	0.30 (0.5)	32.63(6.47)	0.26 (0.13)	5.42 (2.23)	3.18 (1.31)	18.85 (2.11)	27.63 (26.76)	1.39 (1.31)	8.75 (2.43)
	Total	Range	< LD-2.40	1.45–61.37	< LD-0.54	1.77–8.54	1.04–5.02	4.66–34.04	4.44–380.68	1.18–5.40	0.26–13.36
		Med	0.60 (1.00)a	31.59 (22.52)c	0.24 (0.16)b	5.31 (1.26)b	3.12 (0.74)b	18.52 (4.32)b	28.92 (21.02)c	2.52 (0.95)c	9.12 (5.70)b

The order of the median level of the top four metallic trace elements in the samples was found to be Fe > Zn > Mn > Cu, Fe > Zn > Cr > Pb, Fe > Zn > Ni > Co and Zn > Fe > Mn > Cu respectively in cereals, tomato, dried fish and peanuts. Similar results were reported by Ertugrul et al.,2008 in mushrooms from Black Sea region in Turkey^26^.

Iron and Zinc were the elements with the highest concentration in the investigated foodstuffs. The iron highest median value (68.80 mg/kg) was observed in dried fish followed by maize (43.09 mg/kg) and peanuts (28.92 mg/kg). Kolmogorov–Smirnov test stated that there is a significant difference on iron content in the investigated food item. It is know that adequate iron in a diet is very important for decreasing the incidence of anemia^27–29^. However, when their intake is excessively elevated, essential metals can produce toxic effects^30^. Zinc is known to be involved in most metabolic pathways in humans; thus, zinc deficiency can lead to loss of appetite, growth retardation, skin changes, and immunological abnormalities. In the investigated foodstuffs, peanuts have the most abundant zinc content (31.59 mg/kg) which was 2 times higher than the concentrations found in cereals. The lowest median concentration was recorded in the tomato samples (4.39 mg/kg) The mean Zn concentration (13.178 mg/kg) in rice reported in Bangladesh^15^ is similar to the median value of zinc content in rice in this study. The content of Zn and Fe in tomato reported by Gebeyehu et al.^31^ were 24.50 mg/kg and 85.10 mg/kg, respectively which were too lower than the median values found in the current study.

Chromium was detected in 72.22% of maize samples, 96.92% of tomato samples while 100% of peanuts, dried fish and rice contained Chromium. The highest concentration (5.40 mg/kg) was found in peanut sample collected in Dakola. Maize has the lowest median concentration (0.08 mg/kg) followed by tomato (0.95 mg/kg). Gebeyehu et al.^31^ in Ethiopia reported mean concentration of chromium in tomato (1.49 mg/kg) higher than the median concentration of tomato samples. Chromium in the diet is of a great importance because it is an essential trace element. It plays a key role on insulin function and lipid metabolism^32,33^. However, high concentrations may cause adverse health effects^34^.

Hundred percent of all the samples contained Cobalt. The cereal, rice (0.88 mg/kg), maize (0.40 mg/kg) and tomato (0.64 mg/kg) have shown less Cobalt content than dried fish (2.45 mg/kg) and peanuts (3.12 mg/kg). Gebeyehu et al.^31^ in their study found in the tomato samples a mean concentration of 0.63 mg/kg which is similar to the median content obtained in this study. Cobalt is essential for human health since it is a part of vitamin B12. Cobalt is also used in the treatment of anemia in pregnant women for red blood cells stimulation. However, exposure to high concentrations of cobalt could lead to lung adverse effects, such as asthma and pneumonia^35^. The IARC has listed cobalt and cobalt compounds within group 2B as possibly humans carcinogenic agents^35^.

Nickel was found in 94.7% of maize, while 100% of other foodstuffs contained Nickel. Peanuts and dried fish have shown the higher contents of 5.31 mg/kg and 4.17 mg/kg. rice (1.5 mg/kg) and tomato (1.09 mg/kg) had similar content and the lowest concentration was observed in maize samples. A mean concentration of Ni at 5.20 mg/kg in fish samples in Bangladesh has been reported by Islam et al.^14^ which is comparable to the current study. Ahmed and Shaheen^15^ and Rahman et al.^36^ reported 0.213 and 0.01 mg/kg respectively the mean concentration of nickel in rice in their study. These concentrations are largely lower than the median concentration of nickel in rice found in this study. Gebeyehu et al.^31^ reported 1.86 mg/kg in tomato which is comparable to the median value found in this study. Nickel is essential in small quantities, but it can endanger human health at high concentrations.

Copper concentrations in samples are ranged from 1.05 to 5.00 mg/kg (rice), from 1.57 to 3.93 mg/kg (maize), from 0.09 to 1.24 mg/kg (tomato), from 0.15 to 0.67 mg/kg (dried fish) and from 0.26 to 16.26 mg/kg (peanuts). The highest median value (9.12 mg/kg) was recorded in peanut sample while the lowest (0.18 mg/kg) was found in dried fish samples. Islam et al.^14^ reported Cu content in fish ranging from 2.02 to 7.37 mg/kg which is higher than the values found in this study.

Copper is known to be both vital and toxic for many biological systems. It plays a role in activation of more than 30 enzymes of which some are involved in the synthesis of the main component of connective tissues called collagen^35^. Copper can also pose public health hazards at high concentrations.

The highest concentration (34.04 mg/kg) of manganese was recorded in peanuts sample collected in Dakola and two samples of maize from Bobo-Dioulasso did not show the presence of manganese. Tomato shown the lowest median concentration (1.50 mg/kg) followed by maize (2.85 mg/kg) and rice (4.48 mg/kg). Manganese has considerable biological significance, it is associated as an enzymatic cofactor in mitochondria, participates in the regulation of cell metabolism, in receptor binding, and signal transduction pathways^37^. It is quite toxic at high doses. Its toxicity to humans manifest through a psychological and neurological disorder, termed as manganism that closely resembles Parkinson’s disease^38^.

Lead is a toxic metal that has no known vital or beneficial effect on organisms, and is bio-accumulative which can cause serious injury to the brain, nervous system, red blood cells, and kidneys to animals and humans^39^. The Inorganic lead compounds are classified as possibly carcinogenic for human^40^. Lead was found in 82.5% of rice sample, 72.22% of maize sample, 74.4% of dried fish samples, 78.69% of tomato sample and 86.45% in peanut samples. The highest concentration (5.80 mg/kg) was recorded in peanut sample from Dakola. Rates of 77.95%, 66.66% and 32.5% of respectively samples of tomato, maize and rice above the maximum limit set by Codex Alimentarius. The mean concentration of lead (3.63 mg/kg) in tomato reported by Gebeyehu et al.^31^ in Ethiopia was higher than the median content found in the tomato sample considered in this study.

Cadmium was detected in 92, 5% in rice samples, 44.44% in maize sample, 91.81% in tomato sample, in 86.45% peanut sample and 76.75% in dried fish sample. The highest concentration (9.00 mg/kg ) was recorded in tomato sample collected in Cinkanse. Peanut has shown the lowest median value (0.24 mg/kg). Codex Alimentarius has set the maximum limit of cadmium in rice (0.4 mg/kg), in other cereal (0.1 mg/kg) and vegetables (0.05 mg/kg). 47.6% and 71.16%, 0% of sample of maize, tomato and rice respectively shown concentration above the maximum limit. The mean concentration (0.088 mg/kg) of Cd in rice reported by Ahmed et al.^15^ was similar to the median value of the Cadmium content in the current study, while the median content of Cadmium in tomato was lower than the value reported by Gebeyehu et al.^31^ (0.56 mg/kg).

Cadmium is a non-essential and toxic element for human: kidney and bones are the critical target organs of Cadmium contamination. IARC has classified Cadmium as a carcinogen^41^.

The large variability observed on the metallic trace elements contents in the different samples may be related to the variability of environmental both field production and selling conditions. Indeed, metal ions in the environment are a big problem for human beings^42^. These elements can most likely be attributed to rapid development, increased traffic emissions into the atmosphere, industrial activities, and to the lack of sophisticated management of wastes^43^, since the foodstuff are collected through the various agricultural zones, stored and sold in different open markets located in the main cities of the country.

### Estimation of daily intake of metallic trace elements

The estimated chronic daily intake (CDI) of the nine investigated trace metals were evaluated according to the average concentration of each metal in each food group and the consumption data of these food items (Table 3). The consumption data of rice (168.8 g/day) and maize (213 g/day) were provided by the National Institute for Statistic and demographic of Burkina Faso^44^ while tomato (0.6 g/day), peanuts (27 g/day), and dried fish (0.06 g/day) consumption data were collected from FAO database^45^.

**Table 3:** Chronic daily intake and contribution to the maximun tolerable daily intake (ug/day).

	MTDI	Rice		Maize		Tomato		Dried fish		Peanut	
		CDI	%MTDI	CDI	%MTDI	CDI	%MTDI	CDI	%MTDI	CDI	%MTDI
Pb	3000^46^	25.32	0.84	66.092	2.20	0.48	0.02	0.072	0.00	16.2	0.54
Zn	60000^47^	2349.696	3.92	2899.52	4.83	2.634	0.00	0.4326	0.00	852.93	1.42
Cd	500^48^	13.504	2.70	0	0.00	0.054	0.01	0.0048	0.00	6.48	1.30
Ni	300^47^	253.2	84.40	147.108	49.04	0.654	0.22	0.2502	0.08	143.37	47.79
Co	NA	148.544	NA	85.28	NA	0.384	NA	0.147	NA	84.24	NA
Mn	2000–5000^49^	756.224	15.12	607.62	12.15	0.9	0.02	0.7992	0.02	500.04	10.00
Fe	NA	2032.352	NA	12,470.068	NA	4.26	NA	3.7488	NA	780.84	NA
Cr	200^48^	334.224	167.11	17.056	8.53	0.57	0.29	0.1122	0.06	68.04	34.02
Cu	30000^48^	506.4	1.69	460.512	1.54	0.312	0.00	0.0108	0.00	246.24	0.82

NA: not applicable.

Due to the higher consumption rate of both rice and maize, these two cereals have shown the biggest contribution of EDIs. The essential metallic trace elements such as iron (2032.35 μg/day–1240.06 μg/day) and zinc (2349.69 μg/day–2899.52 μg/day) had the higher EDIs followed by manganese (607.62 μg/day–756.22 μg/day). However Chromium had shown higher contribution rate to the maximum daily intake of 167.11%, 34%, 2% and 8.53% for rice, maize and peanut respectively. This higher contribution was followed by Nickel with rice (84.40%), maize (49.04%) and peanut (47.79%). The contribution of rice (0.84%) tomato (0.02%) dried fish (0.00%) and peanut (0.54%) to the MTDI of Lead were less than 1%. According to a total diet study made by the Catalan Food Safety Agency, fish and shellfish account for 12.4% of total Pb intake for the Catalonia population^50^.

Among the food group, rice (2.70%) provided more Cadmium followed by peanut (1.30%) while maize, tomato and dried fish were contributed poorly. It has been reported by the Spanish Food Safety and Nutrition Agency that fish and shellfish only represent a fraction of the total Cadmium intake in the diet, which has been estimated to be between 17.3 and 33.9%^50^.

### Non-carcinogenic risk assessment

**Figure 1:**
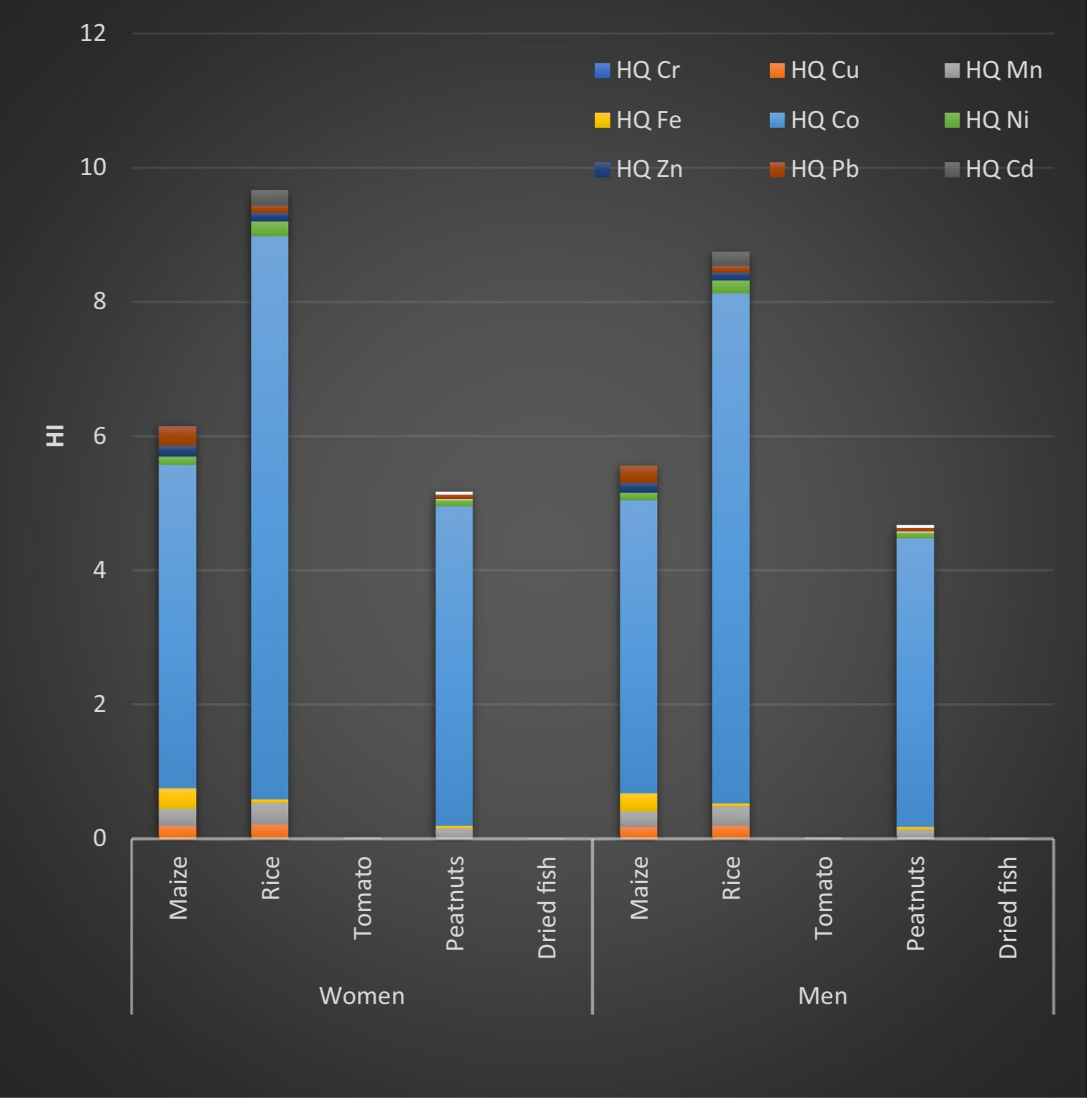
Non-cancer risk index.

The non-cancer risks were expressed as the cumulative hazard index (HI) which was the sum of the individual metal hazard quotient (HQ) (Fig. 1).

The HI values recorded on tomato and dried fish consumption of both men and women varied from 0.009 to 0.23. These values were below the reference value (HI = 1). Consequently, there was no non-cancer risk recorded at this level of consumption of these food items.

However, non-cancer risk situations have been observed on rice, maize and peanuts consumption. Rice was the food item with highest HI value 9.66 and 8.74, respectively recorded for women and men. This was followed by maize (6.14–5.55) and peanut (5.16–4.67). In their study, Nuapia et al. 2017, have reported that in Johannesburg and Kinshasa, the combined HI values were greater than 1 for all the food samples including cabbage, bean, beef and fish for both men and women. These results indicate high potential risk to the local consumers in Kinshasa and Johannesburg via consumption of the food sold in the open markets^8^.

Cobalt was the main contributor to the risk index. The HQ of Cobalt contributed for about for 85% of Hi. This high contribution is related to its concentrations in the samples but especially to its risk oral reference dose (RfD) of 0.0003 mg kg^−1^ day^−1^. Fe and Zn have shown the highest daily intakes, but their contributions to the risk indices were very low. This is due to their higher RfD because of their essential status.

### Carcinogenic risk assessment

**Figure 2:**
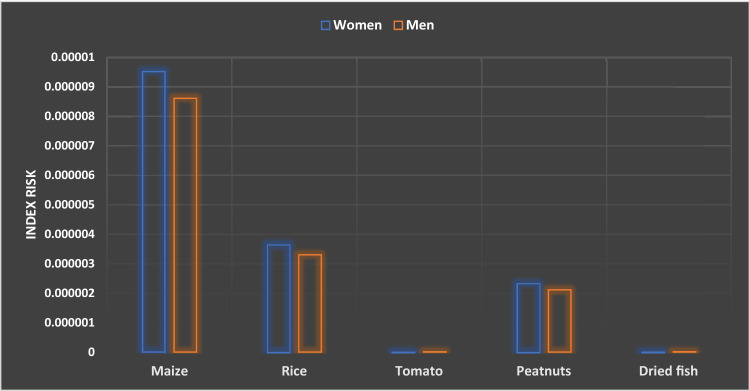
Cancer index risk.

The results obtained (Fig. 2) show the index risk varying from 3 × 10^−6^ to 8 × 10^−9^. None of the index risk values was above the threshold set by US-EPA (IR > 10 − 4); consequently, the consumption of these food groups in Burkina Faso was free of cancer risk from lead contamination.

## Conclusion

This study was consisted to evaluate metallic trace elements contents in some major raw foodstuffs in Burkina Faso and assess the health risk. Metallic trace elements including Fe, Zn, Mn, Co, Cd, Pb, Cu, Ni, Cr were investigated in maize, rice, tomato, peanuts and dried fish. The obtained results showed that the investigated foodstuffs contained an appreciable among of essential trace elements since these food staples are higher contributing to the daily intake. Iron and zinc had the higher EDIs followed by manganese through the consumption of cereal. The non-essential traces elements were found in large portion with more than half of the samples having lead and cadmium concentrations above the limits set by the Codex Alimentarius. The estimation of the daily intake revealed that the population of Burkina Faso are exposed to a non-cancer risk linked to metallic trace elements associated to rice maize and peanut consumption. However, the cancer risk was not a concern.

## Data Availability

The data and materials are available from the corresponding author on reasonable request.
